# Mr. Dunning, on the Influenza

**Published:** 1803-08-01

**Authors:** Richard Dunning


### *To the Editors of the Medical and Physical Journal.*

GENTLEMEN,

THE annexed letter contains some Observations on the late Influenza, in answer to a letter received from Mr. Aikin, Secretary of the London Medical Society. As the paper is much too long to coincide with the plan of the Society on this occasion, I have to request you will give it a place in your valuable Journal

I am, &c.

*June* 11, 1803. RICHARD DUNNING.

---

I beg leave to state to you generally, in reply to your comprehensive queries relative to the late epidemic, that I do not recollect to have observed a case of it distinctly marked in this town, until the 16th or 17th of March; that it prevailed in its highest activity in the course of a few days; and that its range, in a way to claim much attention, was little more than three weeks. Relapses, however, either from incautious exposure, or other inattentions, were not infrequent; but I do not believe that any original seizures of influenza were met with after three weeks; if any did occur, these were so much shaded and lost in other complaints, that they could not be satisfactorily enough ascertained to enable me to speak to them A most petulant and very often an unexpectorating cough, accompanied with uncommon debility, and other effects of the late impression, continued much longer, and in many subjects of it continue to this day; but the influenza had taken such compleat possession of our minds, that it is only very lately that we have ceased to associate the idea of it with almost every indisposition that has since occurred.

Superadded to the usual symptoms of winter catarrh, were a violent pain of the head, *generally of the forehead*, confined over *the orbits of the eyes* along *the frontal sinuses*; sometimes the pain was in the hinder part, but this was rarely; sometimes across the forehead, apparently along the course of the coronary suture; frequently a pain equally violent, of the back, and now and then about the chest and seat of the stomach. In many, a soreness amounting often to much pain, so universally through the muscles, particularly of the extremities, it led sometimes to a remark among medical men, that the seat of influenza seemed often to be muscular—a degree of *peculiar debility* (which was undoubtedly the pathognomonic trait of this epidemic) much greater and more sudden than could by any means be accounted for, either from the violence, length, or general character of the symptoms. The tongue was invariably covered more or less, generally indeed slightly, with a whitish coat; the loss of appetite was entire in most instances. The tenor of the pulse was always much more that of quickness than strength, and there was often a great tendency to perspiration over the whole surface, and in a few cases, a very distressing vomiting continued many days in spite of every remedy. In these instances, perhaps the principle of disease had passed into the stomach, either with the food or saliva, or this symptom more probably was merely sympathetic; certainly, it occurred only in very irritable habits. A nice observer assured me, and my own recollection confirms the remark, that when the head and chest were less affected, the extremities suffered proportionably more, and vice versa. However great the affection of the chest, I never adverted to bleeding, the remarkable prostration of strength always forbade it. If early called, I usually gave an emetic, which I always thought useful, and soon after its operation, a gentle laxative of calomel and rhubarb; these were followed by frequent doses of nitre, liquorice powder, antimonial powder, and Mindererus's spirit, and I generally gave an opiate at night. The latter did not certainly in my practice succeed at first in the early stages of the complaint; but I soon gave them, however, with considerable benefit when joined with a saline camphorated draught; indeed, in all the cases which were more remarkably attended with that peculiar debility that has, I believe, invariably characterized every recurrence of influenza, I gave the camphorated julep and spirit of Mindererus with very satisfactory effects; and after the removal of the febrile symptoms, I have preferred camphor and the squill pill to any preparation of the Peruvian bark. In a few of the severer cases which fell under my care, the repeated application of blisters seemed necessary and useful; indeed, I often thought it one of the best remedies.

On the authority of Dr. Huxham, I constantly recommended a liberal use of wine whey. This admirable combination of wine and milk, (at this time perhaps too much neglected) seemed to me excellently adapted to meet the general indication, for whilst it readily pervades the system or.

or passes off through the great emunctories of the skin and kidnies, it affords at the same time an agreeable bland cordial principle of nutriment, peculiarly well calculated, without heating, to support the constitution under the pressure of so much debility. But after all, little as we know of the real nature and source of the late epidemic, and little fatal as it was in this neighbourhood, I am not sure that the greater number of my patients would not have recovered from it as readily and as perfectly by a little confinement and simple diluent food without medicine at all. Active medicines, and indeed every medicine, with some persons, most frequently nauseate; and hence, no doubt, often disturb and interrupt those agencies of the system, which under the slighter derangements of it at least, would soon determine health. The present anxious and very laudable endeavours to obtain accurate information, with a view to establish some data to be referred to hereafter with confidence in the event of similar epidemics, sufficiently confirm the late indecision of public opinion with respect to the late influenza, and justify an apprehension that medicines must have been sometimes given, and other means of cure adopted, in some measure, at random, and it may be feared now and then even with disadavantage.

"Res fuit hercle diætæ potius accuratæ ac debiti

"Regiminis quam medicinæ elaboratæ."

A few elderly persons only, and these were tottering under previous indisposition, generally of pulmonary, but sometimes of dyspeptic character, were destroyed here. We hear, therefore, with great surprize of its fatality in many other places. Since the influenza disappeared, I do not know that any disorders have prevailed at all remarkably; a few genuine inflammatory cases have occurred, but these have hitherto been certainly less numerous than they frequently are between spring and summer. A fine young woman, who had long enjoyed good health previously to an attack of influenza, is at this time, I much fear, on the brink of phthisis, perhaps it is already formed. It does occur to me, that I do not know an artrhitic friend in this town who has not, within the last two months, been at least once confined; and I have now under my care, an old lady above sixty, whose health has always been infirm, and where I should least of all have expected gout, suffering under a severe first fit of it, and I am in attendance also at this time on several cases of acute rheumatism.

With respect to the much-disputed question, whether the late influenza was or was not a contagious disease, I do not hesitate to say, that the result of my own observations never inclined me to subscribe the former opinion. This singular affection appears to have gone on in a pretty regular progression from east to west. We heard of it at first on the Continent; after some time in London; in a given time it seems to have reached Exeter, and from thence to Plymouth; and we now hear of it in Dublin; and, which is a very remarkable circumstance in its history, it does not appear that the course of it has been materially interrupted or forwarded by the winds. Its attack was here so sudden that it reached its acmè in a few days, and its retreat, I believe, was equally sudden with respect to new seizures.

The first well-marked cases of it which fell under my observation, were a woman and her two sons, fourteen and sixteen years old; these three were attacked within twenty-four hours of each other. Two days after I was called to a family consisting of six, a man, his wife, and four children, all of whom were taken ill nearly at the same time; two or three of them were previously very weakly, and all of constitutions extremely delicate. The aspect of the house in which they lived was due north. From this time my calls were continual from morning till night. I visited many families that were seized pretty regularly in succession in a way that might strongly persuade to a belief of contagion; many families in which several or all were seized nearly at once, and many where a single person only was affected. Indeed, I suffered in my own person no inconsiderable portion of influenza; and although I was occanally in the midst of a large family, and my children perpetually about me, none of them to my knowledge felt a shade of it.

My ingenious neighbour, Mr. Little, (who thinks exactly with me on this occasion, as far as relates to contagion) whose practice is probably more extensive than that of any other medical man in this town, has told me, that availing himself of an opportunity when he was less professionally engaged, he visited some friends at Exeter, on the 13th of March; that he found the influenza there at its height, and that on his return to Dock, at the end of a week only, he learnt with surprise that we had been fully engaged in it for several days.

Dr. Remmett has very obligingly favoured me with the copy of a letter sent by him to Dr. Duncan, at Edinburgh, in in 1782, in answer to some queries relative to the influenza that prevailed in that year. This letter was either not received by Dr. Duncan, or by some inadvertence or other was not printed in the annals of that year, with the other papers on the same subject. Dr. Remmett never troubled himself, however, to enquire how this happened. The letter is immediately ad rem, and important in many respects, I am therefore very glad to avail myself of it. Such an opportunity of marking the progress of a similar epidemic must necessarily very rarely happen to an eminent physician.. Those who best know Dr. Remmett's professional abilities, and have often availed themselves of his efficient prescription, will not read the following remarks with cursory attention. I should premise that the doctor has never believed these influenzas to have been at all contagious.

"Sir,

"I have received your queries relative to the late influenza, and from the circumstance of having travelled from hence to London (about 212 miles), and back again in five days, I can give you a pretty exact account of its progress. When I left home on Sunday, May 26, it had not then made its appearance among us, nor did I hear of it on my journey, until my arrival in London, on Tuesday evening, when it was so general there, that an apothecary, who was indeed a man of considerable practice, assured a friend of mine, that he had seen near 200 people ill of it on that day. I slept but one night in London, and on my return home, Friday evening, May 31, I began to feel a lassitude, which I at first attributed to the fatigue of an anxious and very hasty journey: On the next morning, however, the other more characteristic symptoms of catarrh came on, together with a feverish habit; and in this state I continued several days, the langour being all that time, and for a week or ten days afterwards, very considerable, but without fever enough to require confinement or take me off from business. I was thus the first person attacked by this very curious disorder in this town, and I think it probable that I brought it with me from London; but it seems to have travelled westward nearly as fast as I did; for on Saturday many were taken down here, and on Sunday, Monday, and the following days many more, and there is no doubt but it made its appearance at Plymouth before it could be discovered at Dock, which is only two miles to the westward of us. What I have said of myself

 applies applies with very little variation to by far the greater part of those who had the disease, which was so general, that I can hardly name half a dozen who escaped it, and they were of habits and modes of life the most opposite to each other, and I have heard of no precautions made use by any person. Even in this first stage of the disorder the fever was, however, in many very considerable, with great heat on the skin, and a state of pulse which induced some practitioners to draw blood, from which I never observed any bad effects, although I do not believe that it was in any case productive of much good in the primary state of the complaint. I should add, that in some the influenza put on the appearance of angina, and in others, it was attended with pains in the limbs, but the catarrhal symptoms were by much the most prevalent form of the disease. Its progress was very rapid for about a week, and after that time it became less frequent, though those who were then affected by it suffered more severely than those who had it at first. I cannot say, that I observed any notable crisis; but I think there were very few, if any, who got through the disorder without some degree of increased perspiration. I know of none who died in this stage of the disorder, and the best remedies were spt. Minder. and the occasional use of gentle laxatives, which were always of much service in reducing the fever, and lessening the uncommon langour with which it was attended. This leads us to account for some *of the most remarkable symptoms*, which may be clearly referred to the biliary organs.— But the most serious part of the history of this disorder is yet to be related as a *sequela*. On the 10th and 11th of June, pleurisies and peripneumonies began to attack those who had not taken proper care of themselves in the first stage of the disorder; or it may perhaps be better to give the name of pleuro-peripneumony to this state of the complaints, for I saw no one in whom the two affections were not combined; and it is worth remarking, that many became extremely yellow during the progress of this part of the disorder. On the 13th and 14th, (the weather being extremely hot) many more were ill of it, and for ten days it was so very prevalent, that it was hardly possible for me to do more business than fell to my lot. Here, however, notwithstanding the languor and other bilious appearances, blood-letting with blisters and the most powerful antiphlogistic regimen became indispensably necessary from the violence of the fever, the oppression of the breast, and acute pains in the side; and the blood was remarkably markably sizy and very much cupped. The loss was not attended, however, with as quick a relief as it generally produces in inflammatory complaints of the breast; but I have never known any alarming epidemic in which I had so much reason to be satisfied in the end with any mode of treatment. All other diseases seem to have been lost for some time in this one epidemic. As to the brutes, I can give no satisfactory information concerning them; but I am inclined to believe, that coughs were then and have since continued very frequent among horses. The difficulty of procuring dissections in private practice, (and mine is entirely of that sort) prevents my saying any thing to you on that head; and with respect to the Peruvian bark, I saw nothing to induce me to try it in either stage of the disorder; but, with convalescents, it has been productive of all its usual good effects.

"R. B. REMMETT, Plymouth."

Dr. Huxham observes, of a similar epidemic in 1733, "Nos tamen Plymuthi ante iv iduum Februarii vix attigit: quo die nempe Saturni, plurimi subito quasi correpti erant, postridie innumeri, ad xv. kalend. Martii omnes undique." The late influenza then, it appears, reached this place from Exeter at the most in three days, and spread through the whole of this large town in two or three days. Dr. Remmett has most unquestionably proved, that the epidemic of 1782, travelled at least with equal celerity, and that it observed the same line of direction; and the memoranda of our Hippocratic Huxham inform us, that the febres catarrhales epidemicæ of the years 1733 and 1737, were most strikingly similar, both in respect to the suddenness of their appearance and general concourse of symptoms. Did ever the most malignant contagious disease on record spread with so much rapidity as these? If these epidemics spread with such unexampled rapidity through the medium of contagion only, might we not, consistently with all former observations, expect that their fatal effects on human life would have borne some proportion to such extreme activity? We can, therefore, more satisfactorily account, I presume, for this sudden dispersion of them through this kingdom, perhaps through Europe, and a considerable portion of the globe, by referring the cause of them to some peculiar constitution of the air, or rather to adventitious miasms or effluvia casually floating and conveyed through it. The latter, it is very possible, may be irregularly diffused through the atmosphere, may

 impinge, impinge, therefore, on some persons in larger masses, and pass others altogether; and hence something like a conjecture may be offered, why some villages may have escaped the late epidemic, and why it has appeared in various degrees and different shades of character; but here too we must take into the account the variety of constitutions.

We have often heard of fatal and contagious fevers prevailing in different large towns of this kingdom, and that although these have been left to themselves, in many instances formerly, as far as relates to any means taken to prevent their spread, yet they have not extended beyond the respective towns, or, at the most, their immediate neighbourhood. The small-pox, of the worst character, are very seldom, if ever, out of these large towns; we hope now however, to be able to report, at no very distant day, their entire extinction in them; and yet I am informed that this very infectious disease has not been conveyed to Modbury during the last eight or ten years, a market town at the distance only of sixteen miles, and between which and Plymouth there is a great and daily intercourse. Indeed, were the plague itself, or any other the most contagious disorder of which we have any mention, at this moment in a central town of this populous kingdom, and the present intercourse with it not in the least suspended, I much doubt, indeed I will venture to say, that I do not believe that either of these would be propagated through this country with half the rapidity (might I not say with a hundredth part the rapidity) and regularity which have marked the progress of the late influenza. On the other hand, I entirely believe, that if all intercourse had been wholly discontinued throughout the kingdom during the last four or five months, that the late epidemic would, notwithstanding, have pervaded it much in the same manner we have seen it. Assuredly, then, this affection has been rather wafted on us, than communicated to us in the way of personal intercourse from town to town. Dr. Remmett tells us, that he travelled with the complaint to Plymouth, and there is no reason at all to doubt the fact; but how can we possibly believe that the infection issued from him alone, and from this source was propagated through this extensive town, containing more than twenty thousand inhabitants, in little more than twenty-four hours? Again, if these epidemics are so actively contagious, why do they not diverge with persons going in all directions?

Why

Why should they, unlike every other contagion of which we have any knowledge, pass regularly on in one unvaried rout?—But it has been often observed during the prevalence or previous to these epidemics, that various animals and birds have been affected and destroyed; and if I am not much mistaken, even an impression on the vegetable creation has been somewhere mentioned. I have just learnt, and I have no doubt the information is perfectly correct, that several horses died in this neighbourhood, very suddenly, during the time the late influenza was at the worst with us. That during the close of the last year, and the early months of this, that horses were every where unusally diseased, that very many died, (I knew a neighbouring farmer who lost three, and with difficulty saved several others) and that such were the apprehensions of the farmers for their horses at this time, it was a practice with those who thought the distemper infectious, to put large patches of tar upon the breasts of them by way of prevention; but many attributed, however, these disorders to the horses having eaten insects, which for many weeks were innumerable, and covered the fields in a most extraordinary manner, wherever there was any length of grass; and this, from the mildnes of the season, was general in almost every field. These were covered with a sort of spider's web, and where-ever you stepped, these insects flew off in vast numbers.—I noticed them many times—they were a long-legged, indeed, a sort of winged spider—I believe of the class Diptera; name, Oleracea. A respectable gentleman farmer, living near Modbury, on whose veracity I have the most perfect reliance, assures me, that towards the end of March last and beginning of April, during the prevalence of the influenza, that many of his horses, and those of his neighbours, were very much disordered: the disease, among them, was called the squinsy, and was marked with the following symptoms: Running at the nostrils, cough, sudden weakness and loss of flesh—(these are his own words)—none died—abscesses frequently formed and broke externally, between the cheek bones, about the root of the tongue, and sometimes internally, about the same situation. The same intelligent person, Mr. Parsons, tells me, that in November and December last, dogs were generally affected with a disease, termed with them the Houst; which he observed, seems to consist of a continual effort to vomit, and that froth and slime were thrown up by these efforts efforts in considerable quantities; many of these animals were ill several weeks, and many died. In the advanced period of this distemper they were very subject to fits, as they call it, running here and there, and into pools and ponds; several ran off and have not been since heard of; they never attempted to bite, and therefore the apprehensions which were at first entertained that they were mad, were soon removed. I have judged this correct information too important to be omitted:—can these general and concomitant effects be attributed on any principle of reasoning, or even of probability, to intercourse and the medium of contagion?

The active sphere of febrile contagion, even that of the plague, is now, I believe, generally acknowledged to be limited to a few yards, if not feet; and, therefore, until we are more acquainted with the laws of contagion, and know what the principle of it is, we may be allowed to assert at present a belief, that the atmosphere, of *all contagions* which have a power of affecting the human constitution, is equally circumscribed; must not then the almost inconceivable progress in this point of view, which we have shewn always marks the influenza, and, I believe, has given it this designation, and also the synchronous affections of various animals, be unquestionably referred to some common agent or cause, at once applicable to all—a peculiar impregnation of the atmosphere? I believe these observations apply, with the strictest analogy, to the late epidemic. Dr. Huxham, adverting to the cause of those which occurred in the former part of the last century, and which, with his usual accuracy, he has most minutely described, or rather, indeed, delineated,—says, "Omnino mihi pendere videtur à crasso, udo, gelidoque aëre, spiracula cutis occludente et acrem intra corpus colluviem congerente; nam præcedit hunc morbum semper, ac comitatur crassa admodum humidaque atmospheræ temperies." I must take liberty, however, to remark here, but truly with the most respectful deference to the opinions of so great a man, that if this state of the atmosphere alone were sufficient to produce epidemics of this description and extent, that these would necessarily have occurred much oftener than we have hitherto met with them; that from our present knowledge of the effects of atmospheric air on the human body, we are obliged to conclude, that no possible combinations of it, depending on dryness, moisture, heat and cold, as these happen in this part of Europe, or, indeed, in any part of the world, would probably occasion such consequences as we have lately seen result from from it on the human constitution. If it follows, that a long continuance of that state of the air just mentioned, shuts up the pores, &c. the effects we might expect from these, would be a morbid plethora, gradually formed, and attended with corresponding relaxation and debility; but these do not constitute influenza, something more indisputably is necessary; more consistently with the present state of science will the materies morbi be sought for, on this and similar occasions, in some decomposition of the other constituents of the air, or be referred to some extraneous effluvia casually floating in it. Have any symptoms, at all corresponding with those of the late epidemic, ever been observed in any person under experiments, or cure, at the Pneumatic Institution?

If it were possible to find persons with hardihood and patriotism enough for the purpose, it is possible, and perhaps not improbable, that the latent principle of the late impression on the human system, would sooner be detected by a long suite of diversified experiments in this institution, under the direction and observation of its celebrated founder, or some ingenious philosophical Davy, than by any other means or inquiries that can be set on foot.

Has this deleterious principle to human health arisen from any particular exhalations of the earth, extensive lakes, forests, or morasses. We know that marsh effluvia constantly produce here epidemics, and that they are not contagious. Mildews, and blights of vegetables, fruits, and even trees, are often, I believe, very correctly referred to animalcules; by these, the entire organization of leaves is very soon completely destroyed, and many a healthy tree have I seen suddenly sicken, linger, and die; this is no infrequent occurrence. The atmosphere, it may be, and I believe is, full of animalcules. Imagination cannot conceive the infinite minuteness of these animated atoms. There is no putrefaction where these are not most abundantly present. A drop of stagnant water is a drop of animals. Nothing forwards, perhaps, the putrescency of animal substances so rapidly as the blow or germ of insects and animalculæ; indeed, they would seem almost to be at once the principle and pabulum of putrefaction. Have then adventitious myriads of those, or any state of our own, had any remote share in producing the late sedative impression on human life? The latest, and most philosophical writers, assure us of a very beautiful depurating process of the atmosphere, depending on, and even constituting stituting one of the most important economies of the vegetable kingdom.

If this be true to the extent it has been asserted, must not any temporary diminution of it, to any considerable extent, from blasted forests, large tracts of vegetation, or other natural causes unknown to us, be followed with a corresponding insalubrity of the air, productive of disease to animal life? Has that deterioration of the atmosphere producing epidemics, like the late influenza, (if they are produced by any state of it) arisen from a plus or minus state of it? Have the cause or causes of influenza, be they what they may, been determined successively from east to west, by any influences of the sun or moon? or to what other physical causes can this rout of them be attributed?*

Has the influenza appeared in the North Sea? If so, at what distance from land, and when? Has it been met with in the Atlantic; when and where?

It has not been disputed, I believe, nor is it probable it will be, that the late epidemic followed immediately, or very shortly after the application of its cause or causes; neither will it be denied, I think, that the application of this was always on the mucous membrane, lining the passages to the lungs, and on their various connections in the nostrils, frontal sinuses, &c.† This idea, the primary and great affections of the chest and head, the cough, gravedo, sneezing, vertigo, and catarrh, tend very much to strengthen.‡

Now, nothing can be more in opposition than some of these remarks are, to many circumstances undeniably established relative to the introduction and effects of most contagious principles on the human body. Typhus fever, as we usually meet with it here, is not formed in less than eight or ten days, in persons exposed to its influence.

---

* Some of these queries and observations, when I had written them, appeared to me, as they undoubtedly are, exceedingly excentric, and I was therefore about to throw them by—but on reconsidering them, it struck me that they might possibly glance remotely at a something, which might bear more immediately on the subject; as, then, they occupy but a very little paper, and a small portion of time, I felt inclined rather to submit to the imputation of *a little wandering*, than suppress them. Bold inquiry and novel conjecture, will be necessary to ascertain the subtle cause of influenza.

† And it may be possible through the inhalents of the skin.

‡ Can the soreness and pain of the limbs be referred to the causes of influenza impinging on the skin?

The

The contagion of the small-pox does not produce its effects in so short a time as this; not sooner, indeed, than in fourteen or twenty days.*

The measles, I believe, are determined by the same laws, and very probably most other contagious principles. Even the syphilitic principle of contagion, applied through the absolute medium of immediate contact, under circumstances of all others the most favourable to its sudden absorption; is many days before it establishes itself even locally, very often does not at all; and sometimes months and years elapse before it gains complete possession of the system. These observations might be much extended.—Dr. Fordyce says, "That when matter does not produce fever immediately, it does not enter the blood vessels, but lodges in some part of the body for some time, and as soon as it enters the blood vessels, and touches the heart, the fever immediately comes on." I quote Dr. Fordyce, because, I believe, that fevers arising from *contagion per se*, do never take place until the principle of them has reached the blood vessels. The principle of contagion (for all contagions may be only modifications of the same principle) is very possibly so wholly extraneous, and so extremely destructive of human life, that were this principle immediately to enter the system as soon as exposed to it, life might always be irresistably extinguished very suddenly, or at the most in a few hours. If this conjecture comes near the truth, Nature it would seem, has kindly provided that the constitution shall not be suddenly surprized, and has therefore given it time to meet the impending enemy with all its resources of self preservation.

We hear, indeed, of persons attacked by contagious fever, and destroyed by it in twenty-four hours, but we never see this happening in this country, at least in this part of it. As such effects of contagious fever, then, are so contrary to any experience we have of it, (for we must reason from what we know) it will not be very difficult to believe, that these instances of sudden mortality can happen only from contagion (if it ever happens from contagion), in its most concentrated state, suddenly introduced in union with, and through the medium of, an atmosphere most highly vitiated and destructive of the principle of life.

---

* It is a very remarkable fact, that the variolous principle applied externally to the skin, establishes itself earlier in the system than when received casually into it.

life. But after all, it is even a dispute with the latest and best writers, whether these terrible diseases are contagious or not. But with respect to the air, we know that this can be so vitiated by a variety of natural and artificial means, that a single inspiration of it immediately extinguishes human life; and we can admit, therefore, very readily, that it can be more or less changed from its healthy standard, so as to produce every possible gradation of effect on the human body, from the slightest indisposition to the most sedative impression and death.

Mr. Young, of Plymouth, a gentleman of the greatest respectability in this neighbourhood, and of much learning and great talent for observation, in a conversation on the subject of influenza, expressed to me, in the strongest terms, his entire disbelief that epidemics of this description are in any degree contagious. To the opinions of this gentleman who, from a very long and very extensive practice from which nothing ever diverted his attention, has had opportutinies of noticing several recurrences of influenza, which he assures me he has minutely observed, I pay the greatest deference.

After I had written the preceding observations, of which Mr. Young has been pleased to speak in terms of approbation, he very kindly pointed out to me the following important quotations from an old author, Petrus Salius Diversus, than which nothing can more conclusively apply to some of the foregoing remarks, and, indeed, to the subject of influenza altogether. These extracts are long, and they might have been with great effect much extended; it is impossible to abridge them.

Speaking of an epidemic that happened in the year 1580, similar in all its character and consequences to these we have seen lately, and during the late century, and referring its cause to some exhalations and state of the atmosphere, Salius observes, in a work printed at Frankfort, 1586, "Cum enim aer levem tantùm corruptionem passus est, tunc epidimios vulgaresque solium, non pestilentes edere consuevit, sicuti anno proxime elapso millesimo quingentesimo octuagesimo factum est, in quo ex corruptione äeris, levi tamen per universam Europam et ad exteras usque nationes vagarunt morbi illi, qui à vulgaribus diverso nomine appellati, unicam tamen formam tantum in omnibus regionibus habuere. Hi accedebant cum febre in aliquibus validâ, in aliquibus levi, cum capitis gravedine; superveniebat distillatio admodum molesta, quæ in thoracem decumbebat; hinc oriebatur tussis vehemens ex quâ

quâ in principio excreabant patientes tenuia et cruda: Sitis ut plurimum pauca vel nulla erat, in appetentia magna, et gustatus ferè abolitio, *notabilis que lassitudo et corporis imbecillitas quiescente febre* subsequatur; febris ut plurimum in quarta et ante quartam terminabat sed tussis in multis per plures et plures dies superdurabat et vix tandem hi concocta expuebant—hi morbi ut plurimum erant solubres,* in sanitatem que omnes terminabant, præter eos qui valetudinarios, vel debiles, vel senes vel qui angusto essent thorace et distillationibus obnoxii, vel infirmos vel eos, qui pravo utebantur victu quique ægritudine ipsâ ausi sunt indiscriminatim et sine ratione vivere." Again, he says further on, "Quoniam autem non prava nec ingens sed levis fuit hæc corruptio aëris, ideo tantum epidimios non pestilentes protulit morbos." The course of this epidemic was, however, from west to east; he says, "Hæc epidemia à partibus occidentalibus ultimâ hyeme incipiens, et motum habens versus orientem continueque à regione in regionem propinquam transmigrans, nec ineunte vere, nec eo procedente nec etiam adventante æstate imo nec sub cane nec etiâm, subsequente autumno unquam à *proprio motu* cessavit — quinimo et tempori frigidiori et calidiori et flante tàm austro, quam Boreâ et pluvioso et sereno cælo peragravit hasce omnes Europæ regiones et omnia loca indiscriminàtim." He adds of this epidemic;—"Quam eandem pravam et perniciosam aëris qualitatem, ultra homines sensere etiam cætera viventia: hinc aves illæ exteras terras statis temporibus adire solent, hâc aëris pravitate incitatæ, has nostras fugientes ad alias regiones ante tempus avolarant, et quæ super arbores nidificant et dormiunt, super ipsam terram et in locis infimis, altiora caventes, nocte degebant. Bruta autem animantia quæ ex herbarum et arborum germinibus ac foliis victitant eadem aversabantur, quoniam hæc sub dio posita ab aëre *circumstanti* aliqualitur infecta erant, quæ omnia pravitatis et corruptionis aëris (quæ tamen fuit levis) indubitata erant indicia et argumenta."

Salius's definition of contagion is, "Nam contagium nihil aliud est, quam aliquorum vel saltèm duorum contactus, quorum unius in alterum, istius contactûs ratione, similis gignatur morbus." I am very desirous to mention, and I have the doctor's permission to do so, that Dr. Magennis, physician

* Solubres is probably an error of the press for salubres or sanabiles.

physician of the Royal Naval Hospital at this place, a gentleman whose manners are as amiable as he is eminent in his profession, entertains the same opinions with respect to the non-contagious character of the late influenza, as those I have advanced. Mr. Sargeant, to whom I have submitted this paper, assures me, that he thinks with me on the question of contagion as applying to the late and former influenzas. Mr. Sargent is the oldest, and I shall not offend any one if I say he is the most respectable surgeon in this town. He is, I believe, the friend and father of us all.

I am just favoured with the following answer to an inquiry how the influenza appeared in the large well known school of the Rev. Mr. Bidlake, a gentleman of considerable reputation in the literary world. "This school consisted at that time of nearly thirty boarders besides day scholars. An usher assists Mr. Bidlake. This gentleman was taken ill of the influenza, and suffered a good deal from it; however, he continued to attend as before in the school, and although constantly with the pupils, particularly the boarders, no disorder was communicated, and the young gentlemen were free from any symptoms of it. Mr. Bidlake was slightly indisposed, and also a female servant. When the usher became a little better, he went with one of the boarders to a cottage Mr. B. has in the country, and although they slept in the same room, and the influenza had not left the former, yet the other remained well."

The following remarks on the sweating sickness come rather out of place, but they did not occur to me before. Of what genus in nosology was the sudor anglicanus? This non-descript appeared for the first time in September, 1485, and although it raged little more than a month, destroyed many thousands. This epidemic broke out again in 1517, and it was said of it then that it carried off the patient in three days; again, for the third and last time, this fatal disorder spread rapidly through the kingdom in 1551.

This epidemic was big with death, and this character and effect of it seem at first strongly to favour the idea that it was an infectious disease; but if we advert to the little facilities of intercourse that then necessarily subsisted throughout the kingdom, and at the same time the extreme rapidity with which it spread through it, this opinion of it is but very feebly supported. The acute Hume, speaking of this dreadful disease, observes, "There raged at that time in London, and other places, a species of malady unknown to any other age or nation, the sweating sickness, which which occasioned the sudden death of great multitudes; though it seemed not to be propagated by any contagious infection, but arose from the general disposition of the air and of the human body. In less than twenty-four hours, the patient commonly died or recovered; but when this pestilence had exerted its fury for a few weeks, it was observed, either from alterations in the air, or from a more proper regimen which had been discovered, to be considerably abated." Have we any medical history of this epidemic? What historian has given us the best account of it?

It would seem perhaps that I am anxious to establish the fact, that influenza is not contagious. No, the very reverse of this is the case; my fears are, that contagion is not the principle of it; for over any highly vitiated state of the atmosphere, or even slight deviations of it from its healthy standard, whether arising from particular decompositions of its constituent principles, or from exhalation, or any physical causes of which probably we are wholly ignorant, we have not, nor can we ever have, any extensive controul; but with respect to the principle of those contagions which act on the human body, my friend, Dr. Jenner, has possibly opened a door which may ultimately lead on to the extinction of contagion altogether: Considered in this point of view, the present discussion becomes important, and ceases immediately to be, what I have heard it termed, merely an abstract and speculative investigation, from which nothing useful to society can possibly result.

Such are the observations which the perusal of your queries have suggested. Unacquainted with the intentions and plan of publication which the Medical Society are about to adopt on the present occasion, I have sent them with all the expedition my avocations would possibly admit of; they will necessarily, carry in some part of them, the marks of haste. I have gone into many of the questions at considerable length, and have given my opinions, and ventured some conjectures, in the most unreserved manner. Although I hold it a duty to be always at my post, ready to throw with cheerfulness my mite into the public treasury when called on, or whenever I judge that I can be useful, I should not certainly have obtruded my opinions on the present occasion, if I had not been favoured with your printed address. The many inaccuracies of this paper, therefore, which have arisen from haste, and other obvious causes, will, I have no doubt, be received with allowance by the candid. From the idle, the illnatured, and the captious, I shall never expect even the semblance of courtesy. If, what I apprehend will be the case, the paper is extended to an inconvenient, length and from this, or any other circumstance, does not coincide with the intentions of the Society, I have to request that you will return it to me as soon as is convenient, or to Dr. Jenner.

I have subjoined a state of the weather, &c. for Februaary and March last, which was given to me by a very accurate and ingenious man, whose instruments I know are very good ; but when we recollect the wonderful powers of the human machine in accommodating itself with impunity to an increase of heat, &c. vastly greater than ever happens in nature, or rather the power of preserving nearly its original temperature under these circumstances, we may undertake, without much presumption, to assert that no variations of the atmosphere, cognizable in this country at least by any instruments we have hitherto employed, can ever be satisfactorily adduced to account for influenza. Other instruments, and other tests, are unquestionably required, to detect and analyse the latent principle. What share had the nervous system in the late affection of influenza?

### STATE OF THE WEATHER.

| | | | | | |
|---|---|---|---|---|---|
| Feb. 1 | 8½<br>Noon<br>10½ | 39,45<br><br>30,35 | N.<br><br>N. | Fair with clouds | 42<br><br>48 |
| 2 | 8½<br>Noon<br>10½ | 30,20<br><br>29,89 | N.<br><br>NW. | Cloudy with few showers | 46<br><br>50 |
| 3 | 8½<br>Noon<br>10½ | 29,80<br><br>30,04 | N.<br><br>N. | Fair with clouds | 46<br><br>54 |
| 4 | 8½<br>Noon<br>10½ | 30,21<br><br>30,34 | NE.<br><br>E. | Fair all day | 44<br><br>48 |
| 5 | 8½<br>Noon<br>10½ | 30,05<br><br>29,98 | NE.<br><br>NE. | Fair all day | 43<br><br>50 |
| 6 | 8½<br>Noon<br>10½ | 29,71<br><br>29,68 | NW.<br><br>N. | Morning cloudy with showers, evening fair | 43<br><br>46 |
| 7 | 8½<br>Noon<br>10½ | 29,67<br><br>29,93 | N.<br><br>N. | Fair all day | 42<br><br>47 |

| | | | | | |
|---|---|---|---|---|---|
| Feb. 8 | 8½<br>Noon<br>10½ | 30,67<br><br>30,26 | N.<br><br>N. | Cloudy most of the day | 41<br><br>45 |
| 9 | 8½<br>Noon<br>10½ | 30,32<br><br>30,47 | N.<br><br>N. | Cloudy most of the day | 39<br><br>53 |
| 10 | 8½<br>Noon<br>10½ | 30,45<br><br>30,56 | N.<br><br>E. | Fair with clouds | 41<br><br>50 |
| 11 | 8½<br>Noon<br>10½ | 30,55<br><br>30,55 | E.<br><br>SE. | Fair all day | 40<br><br>49 |
| 12 | 8½<br>Noon<br>10½ | 30,47<br><br>30,33 | E.<br><br>W. | Cloudy all day with little rain | 43<br><br>54 |
| 13 | 8<br>Noon<br>10½ | 30,06<br><br>29,98 | W.<br><br>N. | Morning hard rain, after-part fair with clouds | 50<br><br>49 |
| 14 | 8½<br>Noon<br>10½ | 29,98<br><br>29,90 | NW.<br><br>NW. | Cloudy most of the day | 46<br><br>52 |
| 15 | 8<br>Noon<br>10½ | 29,68<br><br>29,56 | NW.<br><br>NW. | Cloudy with showers, wind fresh | 49<br><br>53 |
| 16 | 8½<br>Noon<br>10½ | 29,66<br><br>29,86 | NW.<br><br>NW. | Fair with clouds and showers, wind fresh | 49<br><br>53 |
| 17 | 8½<br>Noon<br>10½ | 29,74<br><br>29,63 | W.<br><br>W. | Rain most of the day, wind fresh | 50<br><br>53 |
| 18 | 8½<br>Noon<br>10½ | 29,67<br><br>29,83 | W.<br><br>NW. | Cloudy with showers | 50<br><br>50 |
| 19 | 8½<br>Noon<br>10½ | 29,70<br><br>29,44 | S.<br><br>S. | Cloudy with showers | 48<br><br>51 |
| 20 | 8½<br>Noon<br>10½ | 29,43<br><br>29,62 | N.<br><br>N. | Morning rain, after-part cloudy, wind fresh | 49<br><br>47 |
| 21 | 8½<br>Noon<br>10½ | 29,80<br><br>30,03 | N.<br><br>N. | Fair with few clouds | 46<br><br>54 |
| 22 | 8½<br>Noon<br>10½ | 30,15<br><br>30,19 | N.<br><br>S. | Fair with clouds | 45<br><br>51 |

| | | | | | |
|---|---|---|---|---|---|
| Feb. 23 | 8½<br>Noon<br>10½ | 30,14<br><br>30,07 | SW.<br><br>SW. | Cloudy with showers | 50<br><br>53 |
| 24 | 8½<br>Noon<br>10½ | 29,94<br><br>30,10 | SW.<br><br>N. | Rain all day, evening fair | 52<br><br>50 |
| 25 | 8½<br>Noon<br>10½ | 30,19<br><br>30,21 | N.<br><br>W. | Cloudy with showers | 46<br><br>53 |
| 26 | 8½<br>Noon<br>10½ | 30,25<br><br>30,23 | W.<br><br>W. | Cloudy all day, with few showers | 51<br><br>55 |
| 27 | 8½<br>Noon<br>10½ | 30,16<br><br>30,41 | W.<br><br>NW. | Morning rain, after-part fair with clouds | 53<br><br>54 |
| 28 | 8½<br>Noon<br>10½ | 30,40<br><br>30,33 | W.<br><br>NW. | Cloudy with small rain most of the day | 50<br><br>56 |
| Mar. 1 | 8½<br>Noon<br>10½ | 30,28<br><br>30,20 | W.<br><br>NW. | Cloudy with small rain most of the day | 53<br><br>57 |
| 2 | 8½<br>Noon<br>10½ | 30,10<br><br>29,55 | NW.<br><br>NW. | Cloudy most of the day, evening hard wind and rain | 53<br><br>55 |
| 3 | 8½<br>Noon<br>10½ | 29,65<br><br>29,87 | N.<br><br>N. | Cloudy with showers of snow | 48<br><br>50 |
| 4 | 8½<br>Noon<br>10½ | 30,03<br><br>30,09 | N.<br><br>N. | Fair with clouds, and few showers of small snow. Fire in the room | 44<br><br>49 |
| 5 | 8½<br>Noon<br>10½ | 29,99<br><br>29,99 | NE.<br><br>S. | Cloudy most of the day with few showers | 42<br><br>53 |
| 6 | 8½<br>Noon<br>10½ | 30,09<br><br>30,23 | E.<br><br>N. | Morning rain, after-part cloudy with showers | 46<br><br>46 |
| 7 | 8½<br>Noon<br>10½ | 30,20<br><br>30,22 | N.<br><br>NE. | Cloudy with few showers | 45<br><br>50 |
| 8 | 8<br>Noon<br>10½ | 30,14<br><br>30,15 | NE.<br><br>E. | Cloudy most of the day | 48<br><br>48 |
| 9 | 8½<br>Noon<br>10½ | 30,07<br><br>30,00 | NE.<br><br>E. | Cloudy all day | 42<br><br>50 |

